# Long-term safety and efficacy of oral bezafibrate use in dogs with hypertriglyceridemia

**DOI:** 10.1093/jvimsj/aalag041

**Published:** 2026-03-12

**Authors:** Marilou Castonguay-Poirier, Lyanne Fifle, Romain Javard, Romain Huvé

**Affiliations:** Internal Medicine Department, DMVet Veterinary Center, Montreal, QC H8T 3R2, Canada; Internal Medicine Department, DMVet Veterinary Center, Montreal, QC H8T 3R2, Canada; Internal Medicine Department, DMVet Veterinary Center, Montreal, QC H8T 3R2, Canada; Internal Medicine Department, DMVet Veterinary Center, Montreal, QC H8T 3R2, Canada

**Keywords:** canine, hyperadrenocorticism, hypothyroidism, peroxisome proliferator-activated receptor, triglyceride

## Abstract

**Background:**

Bezafibrate (BZF) is effective for the treatment of hypertriglyceridemia in dogs, but there is limited data on its long-term use.

**Hypothesis/Objectives:**

Assess the long-term safety and efficacy of BZF in controlling primary and secondary hypertriglyceridemia in dogs.

**Animals:**

Fifty-five client-owned dogs with hypertriglyceridemia.

**Methods:**

Retrospective study. Dogs were treated with BZF once daily at a median initial dosage of 5.5 mg/kg (range, 3.6-11.6 mg/kg) and classified into 3 groups: primary hypertriglyceridemia (group 1), secondary hypertriglyceridemia without changes in treatment for the underlying condition over time (group 2a) or with changes in treatment for the underlying condition over time (group 2b). Serum triglyceride (TG) concentration, and creatine kinase (CK) and alanine aminotransferase (ALT) activities were recorded before treatment (T0) and at subsequent follow-ups (1, 3, 6, 12, and >18 months, as available). Treatment response was classified as adequate (TG decreased by ≥50 % T0) or inadequate (TG decreased by <50% T0).

**Results:**

All groups showed a significant decrease in TG concentration between baseline (T0) and the last available result (*P* <.01). No significant differences in the last follow-up TG concentration were observed among the 3 groups (*P* = .13). The median TG decrease across all groups during the study period was 85%. Adverse gastrointestinal or hepatic effects, possibly attributable to BZF, were observed in 4/55 dogs.

**Conclusions and clinical importance:**

Long-term use of BZF proved safe and effective for most dogs with primary and secondary hypertriglyceridemia.

## Introduction

Hypertriglyceridemia refers to an increase in serum triglyceride (TG) concentration.^1^^,^^2^ This condition is commonly seen in dogs and can be either primary or secondary to other diseases. Hypertriglyceridemia is most commonly secondary and can have various causes such as endocrine disorders (eg, hypothyroidism, diabetes mellitus, hyperadrenocorticism), administration of drugs (eg, glucocorticoids, phenobarbital), pancreatitis, cholestasis, protein-losing nephropathy, and obesity, among others.^2^^,^^3^ In a recent study, secondary hypertriglyceridemia associated with diabetes mellitus was proposed as a link between pancreatitis and diabetes mellitus.^4^

Hypertriglyceridemia typically remains subclinical in most patients. However, owing to its associations with diseases including pancreatitis, insulin resistance, gallbladder mucocele, seizures, and atherosclerotic disease, medical management of hypertriglyceridemia usually is recommended.^5–16^ The mainstay of hypertriglyceridemia treatment is dietary fat restriction and treatment of potential underlying diseases.^3^ In addition to a low-fat diet, omega-3 fatty acids are widely used by veterinarians. A recent study showed a significant reduction in plasma total cholesterol and TG concentrations in Schnauzer dogs with primary hyperlipidemia when omega-3 was added to a low-fat or moderate-fat diet.^17^ However, normalization of TG may not be achieved with dietary fat restriction and omega-3 only, especially in severe hypertriglyceridemia.^18^ In such circumstances, consideration of a lipid-lowering drug is warranted, and fibrates are commonly recommended for the management of moderate-to-severe hyperlipidemia in people.^19–21^ The effects of fibrates are mediated by peroxisome proliferator-activated receptor alpha (PPAR-α), which includes the induction of hepatic fatty acid uptake, a decrease in hepatic triglyceride production, an increase in activity of lipoprotein lipase, an increase in removal of low-density lipoprotein cholesterol (LDL-C) from blood, and an increase in hepatic high-density lipoprotein cholesterol (HDL-C) production.^19^^,^^22^ In human patients, fibrates are generally effective and well-tolerated. Some adverse effects have been reported, including abdominal and muscle pain, vomiting, diarrhea, increases in alanine aminotransferase (ALT) or creatine kinase (CK) activities, and increased risk of cholelithiasis.^20–22^

The hypolipidemic response to bezafibrate (BZF) in dogs, coupled with its short-term safety, has been validated recently in a prospective clinical trial.^23^ However, documentation is lacking regarding the prolonged use beyond 30 days. Therefore, the primary aim of our retrospective study was to describe the long-term safety and efficacy of BZF in controlling primary and secondary hypertriglyceridemia in dogs.

## Materials and methods

### Case selection and data collection

Cases were identified by searching the keyword “bezafibrate” in the medical records of dogs presented at our private referral hospitals (DMVet Veterinary Center, Montreal, Quebec, Canada) between 2016 and 2023. Dogs prescribed BZF to treat clinically relevant hypertriglyceridemia (ie, TG > 221 mg/dL) were included in the study. Treatment response was classified as adequate (TG decreased by *>*50 % T0) or inadequate (TG decreased by <50% T0). The 50% reduction target was extrapolated from studies evaluating the risk factors of pancreatitis and proteinuria in Miniature Schnauzers,^8^^,^^11^^,^^14^ the Canadian Cardiovascular Society guidelines for the management of dyslipidemia,^24^ and the American College of Veterinary Internal Medicine consensus statement on proteinuria management.^25^ More recent evidence in humans suggests a target of <200 mg/dL to prevent recurrent episodes of acute pancreatitis.^26^^,^^27^ Consistent with this therapeutic goal, dogs were excluded if the baseline TG concentration was <221 mg/dL.

**Figure 1:**
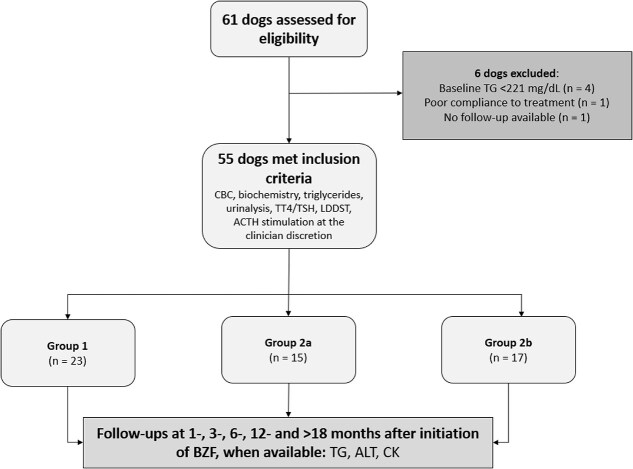
Flow diagram illustrating case selection and overview of the study. Abbreviations: Group = primary hypertriglyceridemia; group 2a = stable secondary hypertriglyceridemia; group 2b = unstable secondary hypertriglyceridemia; ALT = alanine aminotransferase; BZF = bezafibrate; CK = creatine kinase; LDDST = low-dose dexamethasone suppression test; TSH = thyroid-stimulating hormone concentration; TG = triglycerides; TT4 = serum total thyroxine concentration.

All dogs included in the study had baseline serum biochemistry and TG measurements performed. All blood tests were performed after at least 12 hours of fasting. Dogs with increased baseline CK or ALT activity were excluded because of the risk of hepatotoxicity and rhabdomyolysis.^20–22^ Dogs were categorized into 3 groups: primary hypertriglyceridemia (group 1), secondary hypertriglyceridemia without changes in treatment for the underlying condition over time (group 2a), and with treatment modifications over time (group 2b) (Figure 1). Dogs were included in group 1 if primary hypertriglyceridemia was established based on the exclusion of diabetes mellitus, hypothyroidism, hyperadrenocorticism, nephrotic syndrome, and glucocorticoid or phenobarbital administration. Dogs without supporting signalment or clinical signs of hyperadrenocorticism (eg, polyphagia, polydipsia, polyuria, panting) and with normal adrenal gland size on abdominal ultrasonography were also included in group 1, even if diagnostic tests to exclude hyperadrenocorticism were not performed. Dogs were included in groups 2a and 2b when an underlying endocrine disorder was diagnosed or if they were receiving glucocorticoids or phenobarbital. Diagnostic tests for these underlying diseases were performed at the discretion of the attending clinician based on clinical presentation. These tests included CBC, urinalysis, low-dose dexamethasone suppression test (LDDST) or ACTH stimulation test, serum total thyroxine and thyroid-stimulating hormone concentrations, and abdominal ultrasonography. Among the secondary groups, dogs were included in group 2a if treatment for the underlying disease remained unchanged over the study period. These treatments consisted of trilostane, levothyroxine, phenobarbital, glucocorticoids (prednisone or prednisolone), and insulin. Dogs treated using a maintenance physiological dosage of glucocorticoids (*<*0.25 mg/kg/d of prednisone) for hypoadrenocorticism^28^ or glucocorticoids for the management of chronic enteropathy were included in group 2a, conditional to the stability of the glucocorticoid dosage. Group 2b consisted of dogs with secondary hypertriglyceridemia, in which changes were made to their therapeutic plan for the underlying condition during the study period.

All dogs were treated with BZF once daily. The starting dose of BZF was at the discretion of the attending clinician. To allow for weight-based dosing, BZF capsules compounded by a pharmaceutical company (Chiron Compounding Pharmacy Inc.) were used for all included animals. Serum TG concentration, and CK and ALT activities were recorded before treatment (T0) and at subsequent follow-ups (1, 3, 6, 12, and >18 months) as available. All biochemical tests were submitted to Idexx Laboratories for analysis (Idexx Laboratories, Biochemical Analyzer Beckman Coulter AU680, Brea, California). Dogs were required to have a follow-up assessment of their serum TG concentration and serum biochemistry profile at least 1 month after initiation of treatment to be included in the study. If BZF was discontinued during the study period, subsequent results were censored from the analysis. Breed, age, sex, current diet, concurrent conditions, and medications were recorded for each dog. Adverse effects were defined according to the drug manufacturer (eg, diarrhea, vomiting, abdominal pain, increased CK and ALT activities).^20–22^

### Statistical analysis

Descriptive data analysis was performed using commercially available software (Microsoft 365 Excel® Data Analysis). The treatment response is expressed as a percentage of decrease in serum TG concentration, whereas biochemical variables (TG, ALT, CK) are presented as mean ± SD, and median (range). The Wilcoxon matched-pairs signed-rank test was used to assess the change from before and after treatment within each group. To examine differences among TG concentrations in the 3 groups before treatment and at the last follow-up, an initial Kruskal-Wallis test was conducted, followed by a post hoc pairwise Dunn's test with a Bonferroni adjustment if the *P*-value of the Kruskal-Wallis test exceeded 0.05. Analysis was performed using Statistics Kingdom software (https://www.statskingdom.com/index.html). Significance level was set at *P* < .05.

## Results

### Study population

Medical records of 61 dogs diagnosed with hypertriglyceridemia and treated with BZF were identified, of which 55 fulfilled the inclusion criteria. Four dogs were excluded because they had TG concentrations below the significance threshold (<221 mg/dL), 1 because of poor treatment compliance, and 1 because no follow-up results were available after initiation of BZF. Of the 55 included dogs, 31/55 (56%) were neutered males and 24/55 (44%) spayed females with a mean (±SD) age of 10 ± 3 years. Breeds represented included Miniature Schnauzer (11), mixed breed (10), Yorkshire Terrier (7), Miniature Poodle (7), Shih Tzu (3), Labrador Retriever (2), Chihuahua (2), Maltese (2), Pomeranian (2) and 1 each of the following: Pekingese, Fox Terrier, American Eskimo, Vizsla, Beagle, Welsh Terrier, Sealyham Terrier, Dachshund, and Miniature Pinscher.

The median initial dosage for BZF was 5.5 mg/kg (range, 3.6-11.6 mg/kg), and the median final dosage remained the same at 5.5 mg/kg (range, 2.2-11.6 mg/kg). The concurrent conditions known to affect triglyceridemia diagnosed in our study were diabetes mellitus (*n* = 11), chronic pancreatitis (*n* = 9), hyperadrenocorticism (*n* = 9), and hypothyroidism (*n* = 8). Concurrent medications received included insulin (detemir, lente, glargine), trilostane, and levothyroxine. Of the dogs included in the study, 42 were fed a commercial or home-cooked low-fat diet, and 7 were fed a hydrolyzed protein diet. Thirteen dogs (24%) were not being fed a low-fat diet. No dietary change was observed during the study period. Thirty-nine dogs were receiving polyunsaturated fatty acid supplements.

### Triglycerides

**Figure 2:**
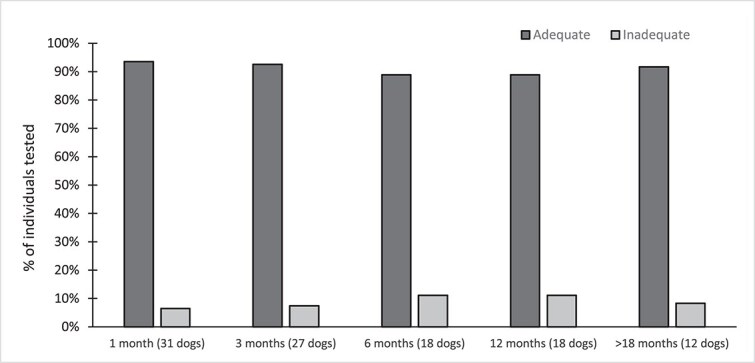
Percentage of all included dogs, across all groups, with adequate (dark gray bars) and inadequate control (light gray bars) after receiving BZF at 1-, 3-, 6-, 12-, and > 18-month follow-ups. Abbreviations: BZF = bezafibrate; TG = triglycerides.

Among all included dogs, the first available follow-up after initiating BZF was at 1 month for 31 of 55 dogs, 3 months for 20 dogs, and 6 months for 4 dogs. One month after the introduction of BZF, an adequate response was observed in 30/31 dogs (97%) and an inadequate response in 1/31 dogs (3%) (Figure 2), with a median decrease in TG concentration of 82%. At the last available follow-up for each dog, 51 dogs (93%) had an adequate response, whereas 4 dogs (7%) had an inadequate response. The median decrease of TG concentration over the study period was 85%, all groups combined.

#### Group 1

Of the 55 dogs in the study, 23 (42%) were included in group 1. The median (range) pre-treatment TG concentration in this group was 594 (244-2085) mg/dL. Because of the retrospective nature of the study, follow-up time points were not standardized for all dogs. Triglyceride concentrations were measured in 11 dogs (*n* = 11/23) 1 month after initiating BZF, all of which had an adequate response. The median TG concentration was 80 (range, 45-323) mg/dL at this time point. Compared with baseline, TG concentration decreased at 1 month post-treatment by a median (range) of 85% (67%-94%). Triglycerides were measured in 14 dogs (*n* = 14/23) 3 months after initiating BZF, and an adequate response was observed in all dogs. The median TG concentration was 81 (range, 52-304) mg/dL, with a median decrease of 81% (range, 57%-96%) from baseline. Five dogs (*n* = 5/23) were evaluated 6 months after starting BZF; 4 (*n* = 4/5) had an adequate response, whereas 1 dog (*n* = 1/5) had an inadequate response. The median TG concentration was 88 (range, 75-480) mg/dL at this time point. Compared with baseline, TG decreased at 6 months post-treatment by a median of 87% (range, 19%-88%). At 12 months after initiating BZF, TG was measured in 6 dogs (*n* = 6/23), all of which had an adequate response. The median TG concentration was 76 (range, 59-193) mg/dL at this time point. The median decrease of TG 12 months post-treatment was 80% (range, 75%-93%) compared with baseline. Five dogs (*n* = 5/23) had follow-up information available >18 months after beginning BZF. An adequate response was documented in all dogs. The median TG concentration was 78 (range, 32-91) mg/dL, with a median decrease of 76% (range, 76%-91%) relative to baseline.

Overall, a significant decrease in triglyceridemia occurred between baseline and the last available TG result in group 1 (*P* <.01). Comparing the change in TG from baseline with the last available follow-up, dogs in group 1 had a median decrease of 85% (range, 19%-96%).

#### Group 2a

Of the 55 dogs in the study, 15 dogs (27%) were included in group 2a. The median (range) pre-treatment TG concentration in this group was 903 (235-2733) mg/dL. Triglycerides were measured in 11 dogs (*n* = 11/15) 1 month after initiating BZF; 10 (*n* = 10/11) had an adequate response, whereas 1 dog (*n* = 1/11) had an inadequate response. The median TG concentration was 173 (range, 72-764) mg/dL at this time point. Compared with baseline, TG decreased at 1 month post-treatment by a median (range) of 79% (+4% to -95%). Triglycerides were measured in 6 dogs (*n* = 6/15) 3 months after starting BZF; 5 of 6 had an adequate response, whereas 1 dog (*n* = 1/6) had an inadequate response. The median TG concentration was 112 (range, 53-161) mg/dL, with a median decrease of 85% (range, 48%-96%) from baseline. Triglycerides were measured in 6 dogs (*n* = 6/15) 6 months after initiating BZF; 5 (*n* = 5/6) had an adequate response, whereas 1 dog (*n* = 1/6) had an inadequate response. The median TG concentration was 119 (range, 57-409) mg/dL at this time point. Compared with baseline, TG decreased at 6 months post-treatment by a median of 91% (range, 37%-98%). At 12 months after initiating BZF, TG concentration was measured in 8 dogs (*n* = 8/15). An adequate response was observed in 6 dogs (*n* = 6/8), whereas an inadequate response was documented in 2 dogs (*n* = 2/8). The median TG concentration was 103 (range, 35-504) mg/dL at this time point. The median decrease of TG 12 months post-treatment was 84% (range, 32%-99%) compared with baseline. Two dogs (*n* = 2/15) had follow-up information available >18 months after beginning BZF. An adequate response was observed in 1 dog, and no response was documented in the other dog. The median TG concentration was 376 (range, 70-682) mg/dL, with a median decrease of 41% (range, 7%-75%) relative to baseline.

Overall, a significant decrease in triglyceridemia occurred between baseline and the last TG result available in group 2a (*P* <.01). Comparing the change in TG from baseline with the last available follow-up, dogs in group 2a had a median decrease of 85% (range, 7%-99%) of TG.

#### Group 2b

Of the 55 dogs in the study, 17 dogs (31%) were included in group 2b. The median (range) pre-treatment TG concentration in this group was 869 (358-3211) mg/dL. Triglycerides were measured in 9 dogs (*n* = 9/17) 1 month after initiating BZF, all of which had an adequate response. The median TG concentration was 180 (range, 98-624) mg/dL at this time point. Compared with baseline, TG decreased at 1 month post-treatment by a median (range) of 82% (75%-97%). Triglycerides were measured in 7 dogs (*n* = 7/17) 3 months after starting BZF; 6 (*n* = 6/7) had an adequate response, whereas 1 dog (*n* = 1/7) had an inadequate response. The median TG concentration was 95 (range, 54-260) mg/dL, with a median decrease of 85% (range, 40%-94%) from baseline. Seven dogs (*n* = 7/17) were evaluated 6 months after starting BZF. An adequate response was documented in all dogs. The median TG concentration was 96 (range, 47-148) mg/dL at this time point. Compared with baseline, TG decreased at 6 months post-treatment by a median of 91% (range, 80%-97%). At 12 months after initiating BZF, TG was measured in 4 dogs (*n* = 4/17), all of which had an adequate response. The median TG concentration was 73 (range, 50-99) mg/dL at this time point. The median decrease of TG 12 months post-treatment was 90% (range, 82%-95%) compared with baseline. Five dogs (*n* = 5/17) had follow-up information available >18 months after beginning BZF. An adequate response was documented in all dogs. The median TG concentration was 107 (range, 87-283) mg/dL, with a median decrease of 87% (range, 81%-97%) relative to baseline.

**Figure 3:**
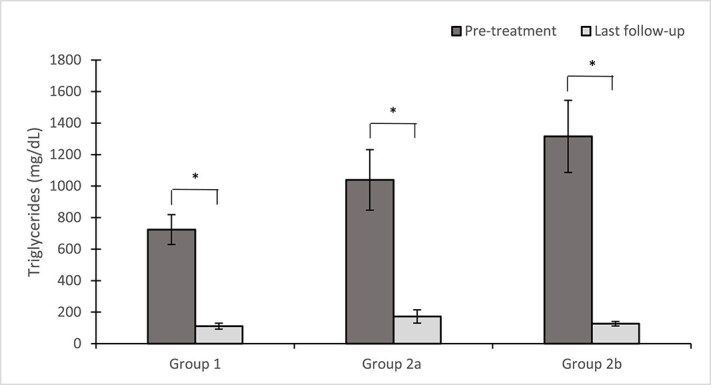
Mean TG concentrations (mg/dL) for each group from baseline and at the last available follow-up after receiving BZF. Error bars indicate standard deviation. *significant difference (*P* <.01).

Significant differences in baseline serum TG concentrations were observed among the 3 groups (*P* = .05). Post hoc analysis showed that baseline triglyceridemia before BZF treatment was higher in group 2b compared with group 1 (*P* = .02), but no difference was observed between group 1 and group 2a and between group 2a and 2b. No significant differences in the last follow-up TG concentration were observed among the 3 groups (*P* = .13) (Figure 3).

### Adverse effects

Of the 55 dogs included, 4 dogs (7%) experienced adverse effects over the study period. These consisted of mild gastrointestinal signs in 2 dogs receiving BZF at dosages of 5.4 and 5 mg/kg, respectively. Of the 2 dogs, vomiting and nausea occurred within 24 hours after the first administration of BZF in one dog. Gastrointestinal signs resolved within 48 hours after discontinuation of the medication. Upon reintroduction of BZF at a decreased dosage (2.7 mg/kg/d compared with the initial 5.4 mg/kg/d) one month later, no adverse effects were observed. The other dog had intermittent diarrhea one week after the initiation of BZF. After treatment cessation, the diarrhea resolved within 48 hours. Subsequently, BZF was resumed at a lower dosage (4 vs 5 mg/kg/d) without adverse effects. A severe episode of hemorrhagic gastroenteritis occurred in another dog 7 weeks after the initiation of BZF at a dosage of 4.7 mg/kg. The medication was temporarily discontinued and reintroduced 5 months later at a lower dosage (4 vs 4.7 mg/kg), and the dog showed no worsening of digestive signs. Given the insufficient response at the lower dosage, the BZF dosage was gradually increased to the initial dosage over 5 months. No further adverse reactions were observed later in this dog. For the last dog, an increase in ALT activity (672 U/L; reference range, 18-121 U/L) was noted 6 months after initiating BZF at a dosage of 5.8 mg/kg. Although ALT activity was within the reference range at the 1- and 3-month follow-ups, a substantial increase was observed at the 6-month assessment. The ALT activity was reevaluated 6 weeks after BZF discontinuation and the introduction of another lipid-lowering drug (chitosan), identifying a 41% decrease in activity. However, hypertriglyceridemia worsened, with concentrations increasing from 483 mg/dL to 1323 mg/dL. Other lipid-lowering drug classes, such as statins, were attempted during subsequent follow-ups without improvement in triglyceridemia. Otherwise, no increase in CK activity occurred in the 3 groups during the study period.

## Discussion

The purpose of our retrospective study was to describe the long-term efficacy and safety of BZF in dogs for the management of primary and secondary hypertriglyceridemia. Adverse effects possibly attributable to BZF were observed in 4/55 dogs (7%). Furthermore, a significant decrease in TG concentration was observed between baseline (T0) and the last measurement in all groups. An adequate response was observed in 30/31 dogs (97%) one month after initiation of BZF, including 11/11 dogs in group 1, 10/11 dogs in group 2a, and 9/9 dogs in group 2b. The difference in adequate response rates among group 1, group 2a, and group 2b at the one-month follow-up could be related to the significantly higher baseline triglyceridemia in group 2b compared with group 1 (*P* = .02), as well as the underlying cause of hypertriglyceridemia. In humans, disease processes such as diabetes mellitus are known to impair optimal response to fenofibrate (FNF).^29^

In our study, the median decrease in TG concentration after one month of treatment was similar among the groups (85%, 85%, and 87%). Our results agree with the results of 3 previous studies evaluating the efficacy of fibrates in dogs with primary and secondary hypertriglyceridemia that reported a median decrease in TG concentration of 84% with BZF, and 88% and 81% with FNF.^23^^,^^30^^,^^31^ The slight differences in the percentage decrease in TG may be explained by the severity of hypertriglyceridemia, which was higher at baseline in our study across all groups (median, 841; range, 285-3490 mg/dL) compared with a previous study (median, 487; range, 350-356 mg/dL).^23^ Our study also included more dogs with secondary hypertriglyceridemia, potentially explaining the suboptimal response to BZF after one month of treatment. Indeed, in a recent prospective study evaluating the safety and efficacy of FNF to control severe hypertriglyceridemia (TG > 300 mg/dL) in dogs, 9 dogs (5 with hypothyroidism and 4 with hyperadrenocorticism) had normalized TG concentrations only after their underlying disease had been treated in combination with FNF, showing the importance of the treatment of the underlying disease in the management of secondary hypertriglyceridemia.^30^ Although the identification and treatment of underlying causes of severe hypertriglyceridemia are considered first, in cases of severe hypertriglyceridemia in humans (TG > 1000 mg/dL), a multimodal approach combining treatment of the underlying cause with TG-lowering agent, such as a fibrate, is indicated to avoid acute pancreatitis.^21^ In our study, TG concentrations decreased significantly between baseline and the last result available in group 2b (*P* <.01), despite suboptimal control of the underlying disease. Moreover, no significant difference was observed in the last follow-up TG concentration among the 3 groups (*P* = .13), reinforcing the efficacy of BZF in the management of hypertriglyceridemia even if the underlying disease is not optimally controlled.

The initial and final doses of BZF used in our study were based on a previous study.^23^ Over the study period, the dose of BZF was increased in only one dog, from group 2a, that had diabetes mellitus. This finding shows that the previously described BZF dosage is appropriate and, in most cases, an adequate response is usually expected.^23^ Our diabetic dog initially was receiving a dosage of 5.2 mg/kg, and given the inadequate response after one month of treatment, the BZF dosage was increased from 5.2 to 6.6 mg/kg, resulting in an adequate response at the 3-month follow-up. This response could be secondary to the increased dosage of BZF. However, it remains possible that a decrease in TG concentration might have been observed during subsequent follow-ups without increasing the dose of BZF. This latter dog had clinically and biologically better control of its diabetes mellitus in subsequent follow-ups without insulin dose modification or any other relevant treatment adjustments, suggesting an effect of hypertriglyceridemia on insulin resistance. In humans, severe hypertriglyceridemia is a known risk factor for insulin resistance, and fibrate treatment, particularly BZF, has been associated with improved glucose control.^22^ Acting as a PPAR-α agonist, BZF also exhibits potent additional activity on peroxisome PPAR gamma. Specific activation of the latter has been associated with enhanced insulin sensitivity during treatment.^22^ In dogs, insulin resistance has been reported in nearly 30% of healthy Miniature Schnauzers with hypertriglyceridemia, based on serum insulin concentration.^10^

A small percentage (5%) of dogs (3/55) developed gastrointestinal signs. Although these clinical signs occurred within a week after initiating BZF in 2 dogs, we cannot completely rule out the contribution of other factors, such as primary gastrointestinal disease or concurrent medications. This uncertainty is further emphasized by the lack of recurrence of gastrointestinal signs when BZF treatment was resumed at a lower dose. Liver toxicity was suspected in one dog because the ALT activity increased more than three times the upper reference range 6 months after starting BZF, whereas it remained within the reference range at the 1- and 3-month follow-ups. Although hypertriglyceridemia can lead to increases in liver enzyme activity,^9^ in this case, TG concentration decreased as ALT activity increased, making hypertriglyceridemia an unlikely explanation for increased ALT activity. Despite extensive clinical use of fibrates in human medicine, the precise mechanisms leading to hepatotoxicity have not been fully elucidated.^22^^,^^32^ The frequency and pattern of hepatic injury caused by fibrates are variable, with manifestations observed within a few weeks or months of initiating treatment or even after more than 6 months or years of treatment, resembling chronic hepatitis and cirrhosis.^32^ Considering the potential for fibrate-associated hepatotoxicity to be manifested several months or even years after treatment initiation in humans, it is plausible that BZF could be the cause of the observed ALT activity increase in this dog. Although a definitive causal relationship remains uncertain, this report emphasizes the importance of regular monitoring of ALT activity in dogs receiving BZF. Additional long-term studies are required to investigate further the potential hepatic effects of prolonged BZF administration in dogs. Only a few case reports describe patients with rhabdomyolysis or myopathy, characterized by diffuse myalgia and muscle weakness, after fibrate exposure in humans.^22^ Myopathy secondary to fibrate treatment also seems infrequent in dogs.^23^^,^^33^ No cases of myopathy or increased CK activity were observed in our study, consistent with a previous study.^23^ However, this observation should be interpreted with caution, because baseline (T0) CK activity and at least one follow-up measurement were available for only 24 of 55 dogs included in our study. Further investigation and a more comprehensive assessment of CK activity in a larger cohort are warranted to confirm the infrequency of myopathy-related complications in dogs undergoing treatment with fibrates.

One dog failed to show a relevant biochemical response after 18 months of BZF treatment. This dog had a prior diagnosis of hypoadrenocorticism, was receiving a physiologic dose of prednisone for a year before BZF initiation, and was fed a hydrolyzed protein diet rather than a low-fat diet because of chronic digestive issues. Although chronic use of corticosteroids may have predisposed this dog to hypertriglyceridemia,^2^^,^^3^ 3 other dogs included in our study, also on physiological doses of glucocorticoids, had an adequate response, suggesting other contributing factors. One possible factor is the lack of dietary fat restriction, a key component in hypertriglyceridemia management.^3^ However, a recent study showed that a lipid-lowering medical treatment, such as a fibrate, is more effective alone in controlling severe hypertriglyceridemia than a low-fat diet, with TG normalization in 85.9% of dogs receiving FNF without fat restriction in their diet compared with 26.6% of dogs solely on a low-fat diet.^30^ Additionally, genetic factors also may be involved, as seen in humans with moderate-to-severe hypertriglyceridemia caused by impairments in TG metabolism by lipoprotein lipase in chylomicrons and very low-density lipoproteins. In these cases of genetic dyslipidemias, fibrates are less effective.^34^ Although such genetic dyslipidemias are poorly characterized in veterinary medicine, they potentially could explain the lack of response in some dogs. Otherwise, the dose and frequency of BZF administration in our study may have been suboptimal for some dogs, because BZF can be administered every 8 or 12 hours in people.^20^

Some dogs may be more refractory to BZF, as opposed to new drugs that are currently under investigation in humans. The fibrate family, encompassing medications such as BZF and FNF, has demonstrated similar efficacy in managing hypertriglyceridemia in dogs.^23^^,^^30^ However, a recent randomized crossover investigation in humans comparing pemafibrate (PMF), a highly selective PPAR-α agonist in the fibrate family, with BZF determined that PMF was more effective in decreasing TG concentrations. Additionally, PMF exhibited a favorable safety profile with respect to liver and renal function when compared with BZF.^35^ Prospective studies are needed to evaluate the safety and efficacy of PMF use in dogs.

Our study had some limitations based on its retrospective design. Endocrine tests were not systematically performed. Consequently, cases of secondary hypertriglyceridemia might have been misclassified as primary hypertriglyceridemia. Moreover, follow-up intervals were not standardized and available at the recommended time points, potentially resulting in missed data on toxicity and adverse effects. The small sample sizes within groups may have limited the statistical power of our study, making it difficult to detect subtle differences. Although BZF dosage was determined based on body weight and prepared in precisely compounded capsules, its content was not verified by analysis. Treatment response was assessed using relative decrease (*>*50%) rather than aiming for normalization of the TG concentration. This approach, however, more accurately reflects clinical practice, where substantial improvement in hypertriglyceridemia has been shown to decrease associated risk factors in both human and veterinary medicine.^8^^,^^11^^,^^14^^,^^26^^,^^27^ Moreover, the absence of dietary standardization and variability in concurrent treatments among the secondary hypertriglyceridemia groups limits evaluation of the efficacy of BZF as monotherapy. It is noteworthy, however, that thirteen dogs (24%) were not being fed a low-fat diet, and 12 of these 13 dogs (92%) exhibited an adequate response based on the last TG measurement. In group 2b, treatments related to the underlying disease were adjusted according to clinical and biological control of the disease. Consequently, the improvement in hypertriglyceridemia in this group might not be due solely to the addition of BZF. Finally, no follow-up abdominal ultrasonography was performed in this cohort to assess the development or progression of cholelithiasis, despite the known increased risk of cholelithiasis (cholesterol composition) with fibrates in humans.^36^^,^^37^ However, this potential adverse effect has not yet been documented in veterinary medicine.

The aim of our study was to describe the long-term safety and efficacy of BZF in controlling primary and secondary hypertriglyceridemia in dogs. Our findings indicate that BZF treatment was associated with a sustained long-term decrease in TG concentrations in dogs, regardless of the underlying cause of hypertriglyceridemia or its management. Overall, adverse effects were uncommon, but regular monitoring of ALT activity and gastrointestinal signs remains advisable. Although these results are promising, additional standardized prospective studies are needed to provide further support and validation for our findings.

## Data Availability

The data underlying this article are available in the article.

## Off-label antimicrobial declaration

Authors declare no off-label use of antimicrobials.

## Institutional animal care and use committee or other approval declaration

Authors declare no institutional animal care and use committee or other approval was needed.

## Abbreviations

ALT: alanine aminotransferase
BZF: bezafibrate
CK: creatine kinase
FNF: fenofibrate
HDL-C: high-density lipoprotein cholesterol
LDDST: low-dose dexamethasone suppression test
LDL-C: low-density lipoprotein cholesterol
PH: primary hypertriglyceridemia
PMF: pemafibrate
PPAR-α: peroxisome proliferator-activated receptor alpha
SHs: secondary hypertriglyceridemia with stable treatment
SHu: secondary hypertriglyceridemia with unstable treatment
TG: triglyceride
